# Kinetochore-microtubule attachment in human cells is regulated by the interaction of a conserved motif of Ska1 with EB1

**DOI:** 10.1016/j.jbc.2022.102853

**Published:** 2022-12-31

**Authors:** Renjith M. Radhakrishnan, Safwa T. Kizhakkeduth, Vishnu M. Nair, Shine Ayyappan, R. Bhagya Lakshmi, Neethu Babu, Anjaly Prasannajith, Kenichi Umeda, Vinesh Vijayan, Noriyuki Kodera, Tapas K. Manna

**Affiliations:** 1 School of Biology, Indian Institute of Science Education and Research, Thiruvananthapuram, Kerala, India; 2 School of Chemistry, Indian Institute of Science Education and Research, Thiruvananthapuram, Kerala, India; 3 Nano Life Science Institute (WPI-Nano LSI), Kanazawa University, Kakuma-machi, Kanazawa, Japan

**Keywords:** microtubule, kinetochore, mitosis, spindle, EB1, Ska1, +TIPs, EB1, end binding 1, EBH, EB homology, FBS, fetal bovine serum, GST, glutathione S- transferase, HS-AFM, high speed atomic force microscopy, KT, kinetochore, MT, microtubule, Ska, spindle and kinetochore associated

## Abstract

The kinetochore establishes the linkage between chromosomes and the spindle microtubule plus ends during mitosis. In vertebrates, the spindle-kinetochore–associated (Ska1,2,3) complex stabilizes kinetochore attachment with the microtubule plus ends, but how Ska is recruited to and stabilized at the kinetochore-microtubule interface is not understood. Here, our results show that interaction of Ska1 with the general microtubule plus end–associated protein EB1 through a conserved motif regulates Ska recruitment to kinetochores in human cells. Ska1 forms a stable complex with EB1 *via* interaction with the motif in its N-terminal disordered loop region. Disruption of this interaction either by deleting or mutating the motif disrupts Ska complex recruitment to kinetochores and induces chromosome alignment defects, but it does not affect Ska complex assembly. Atomic-force microscopy imaging revealed that Ska1 is anchored to the C-terminal region of the EB1 dimer through its loop and thereby promotes formation of extended structures. Furthermore, our NMR data showed that the Ska1 motif binds to the residues in EB1 that are the binding sites of other plus end targeting proteins that are recruited to microtubules by EB1 through a similar conserved motif. Collectively, our results demonstrate that EB1-mediated Ska1 recruitment onto the microtubule serves as a general mechanism for the formation of vertebrate kinetochore-microtubule attachments and metaphase chromosome alignment.

Faithful chromosome segregation requires the formation of physical linkage between the spindle microtubule (MT) plus ends and the kinetochore (KT), a supramolecular structure composed of ∼ 100 proteins assembled on the chromosomal DNA (1, 2, 3). For segregation to commence, the sister chromatid KTs need to achieve bioriented end-on attachment with the plus ends of the KT-targeting MTs (4, 5, 6, 7). However, since the plus ends are highly dynamic due to their inherent nature of rapid polymerization and depolymerization, for an efficient end-on attachment to establish and withstand, the plus end association of the outer KT needs to be stabilized and it should occur with high specificity (8, 9, 10, 11). Though a number of outer KT and spindle MT-associated proteins have been known to be associated in mediating KT-MT attachments (1, 2, 3), the mechanisms how the KT end-on attachment is established and stabilized is yet not clearly defined.

In *Saccharomyces cerevisiae*, hetero-decameric complex Dam1/DASH maintains KT-MT attachment by simultaneously binding to MTs and interacting with the outer KT protein NDC80 (3, 12, 13, 14). Because Dam1 forms oligomeric ring structures on the MTs *in vitro* and the rings can slide along the purified MTs, it is believed that Dam1 ring can stabilize KT attachment to the dynamic MT plus ends *in vivo* (15, 16, 17). In higher eukaryotes, an analogous function is mediated by Ska (spindle and KT associated) complex, which consists of Ska1, Ska2, and Ska3 (18). Ska is essential for chromosome alignments and KT-MT attachments in vertebrate cells (19, 20, 21, 22). Ska is thought to couple KT attachment with the dynamic MTs since it can associate with KTs through interaction of Ska1 and Ska3 with NDC80 in one side and on the other side, it can associate with MTs through Ska1 (5, 23, 24, 25, 26, 27, 28). Furthermore, Cdk1-mediated phosphorylation of Ska3 plays an essential role in Ska interaction with Ndc80 and thereby its recruitment to the KT (26, 29).Though both Ska1 and Dam1 complex can independently bind to the MTs *in vitro*, their localizations *in vivo* are mostly confined to the KT-MT junction, but very weakly on the spindle MTs (5, 15, 23, 30, 31, 32), suggesting of other mechanisms that recruit Ska1 to the KTs. Supportively, recent studies indicated that KT localization of Ska in human cells/Dam1 in yeast is regulated by the general MT plus end (+TIP) associated protein, EB1/Bim1 (31, 33). EB1 is the central regulator of dynamic +TIPs (plus tip tracking proteins) network. It recruits numerous structurally and functionally diverse +TIPs to MT plus ends through direct interaction (34). In human cells, EB1 facilitates chromosome alignment by stabilizing KT localization of the Ska complex (31, 35). However, the mechanism how Ska recruitment to KTs is regulated by EB1 remains to be understood.

Here, we show that interaction of EB1 to a disordered loop region of Ska1 located in its N-terminus is essential for KT recruitment of Ska complex and metaphase chromosome alignment in human cells. Atomic force microscopy analyses reveal involvement of the Ska1 loop in mediating EB1-Ska1 binding and leading to the formation of a complex with defined structure. A conserved motif in the disordered loop of Ska1 is primarily involved in Ska1-EB1 binding and metaphase chromosome alignment. The EB1-binding Ska1 motif bears close similarity with the Serine-any amino acid-Isoleucine-Proline (SXIP) motif of other EB1-binding +TIPs, which mediate their MT recruitment in EB1-dependent manner (33, 36, 37, 38, 39, 40). Our NMR data further showed that the Ska1 motif-binding affects those residues in EB1, which serve as the binding sites of SXIP motifs of a few distinct +TIPs (36, 41, 42). The results demonstrate that Ska stabilization at the KT-MT interface is mediated by Ska1 binding to EB1 through its disordered loop region and primarily, through a conserved motif in the loop. The findings also implicate that formation of KT-MT attachment is facilitated by recognition of EB1 protein by Ska1 at the KT MT plus ends and it involves a mechanism analogous to the EB1-binding +TIPs.

## Results

### Ska1 N-terminal–disordered loop is essential for chromosome alignment and KT localization of Ska1

**Figure 1:**
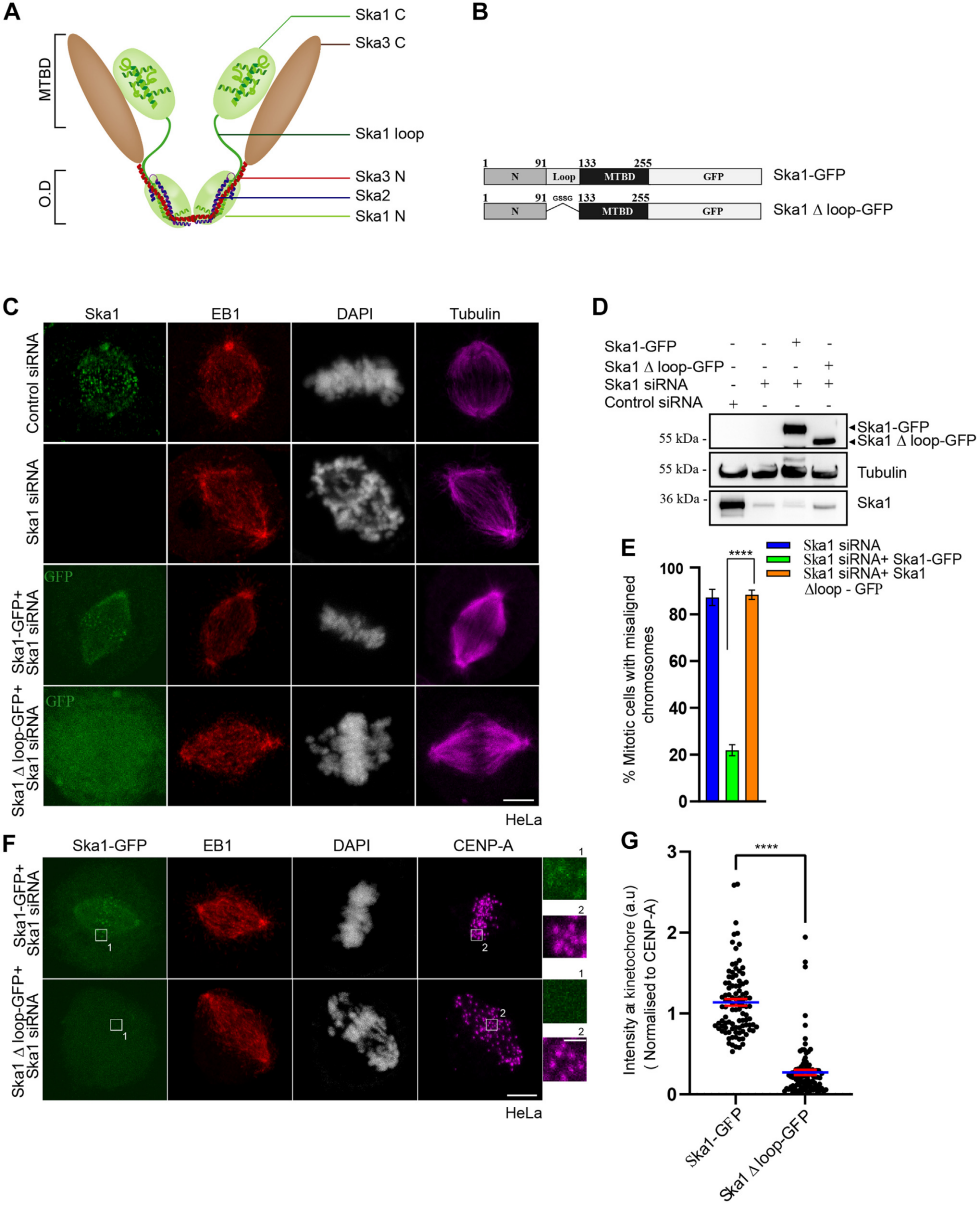
**Kinetochore recruitment of Ska1 involves its N terminal disordered loop.***A*, cartoon representation of Ska complex showing the domains of individual Ska proteins (Ska1, Ska2, and Ska3). Ska1, 2, 3 are labeled with different colors. Microtubule-binding C-terminal domains of Ska1 and Ska3 are labeled as MTBD. The N-terminal oligomerization domain is labeled as OD. *B*, schematic representation of GFP-tagged full length Ska1 and Ska1 Δ loop constructs. *C*, representative immunofluorescence confocal microscopy images of HeLa cells transfected with control siRNA, Ska1 siRNA, Ska1 siRNA+ siRNA-resistant Ska1-GFP (48 h), and Ska1 siRNA+ siRNA-resistant Ska1 Δloop-GFP (48 h). Control and Ska1 siRNA only–treated cells were immunostained with rabbit polyclonal Ska1 antibody (*green*), and EB1 was stained with rat monoclonal EB1 antibody (*red*). Ska1-GFP or Ska1 Δloop-GFP–expressed cells were stained with polyclonal EB1 rabbit antibody. The GFP channels were imaged directly. Microtubules were stained with α-tubulin mouse monoclonal antibody in all cases (*violet*). DNA was stained with DAPI (shown in *white*). Scale bar represents 5 μm. *D*, Western blot images of cell lysates of Ska1 siRNA-, Ska1 siRNA + Ska1-GFP, and Ska1 siRNA + Ska1 Δloop-GFP–treated cells showing expression levels of the exogenous Ska1 proteins with simultaneous depletion of endogenous Ska1. *E*, plot showing percentage of mitotic cells with misaligned chromosomes in Ska1 siRNA-, Ska1 siRNA + Ska1-GFP-, and Ska1 siRNA + Ska1 Δloop-GFP–treated conditions. Data are mean ± SEM. ∗∗∗∗ represents *p*< 0.0001. *F*, HeLa cells in Ska1 siRNA + Ska1-GFP or Ska1 siRNA + Ska1 Δloop-GFP–treated condition were imaged for localization of the GFP-fused Ska1 proteins at the kinetochore (KT). Insets 1 and 2 represent GFP-tagged Ska1 proteins and CENP-A, respectively. EB1 was stained with rabbit EB1 antibody, and CENP-A was stained with mouse monoclonal CENP-A antibody. DNA was stained with DAPI. The scale bars in the main and inset figures are 5 μm and 1 μm, respectively. *G*, plot showing the intensity of Ska1-GFP *versus* Ska1 Δloop-GFP at individual KTs in HeLa cells. Data are mean ± SEM. ∗∗∗∗*p*< 0.0001 (n = 3). Approximately, 100 KTs in each of the three experiments were measured.

Ska1 consists of two structural domains, globular C-terminal domain (residues 133-255), which possesses MT-binding ability (5), and an N-terminal (1-91) helical domain (Fig. 1*A*) (43), which interacts with other components in the Ska complex. These two domains are connected by 40-amino acids–disordered loop region, with residues (92-132). Deletion of Ska1 loop causes delay in anaphase progression in human cells, though the loop-deleted Ska1 can bind to MTs *in vitro* similarly as the full-length protein (43), suggesting that Ska1 loop has a distinct role during mitosis progression and that is presumably independent of the MT-binding activity of Ska1 mediated by its C-terminus. We therefore aimed to characterize the mitotic defects resulted in the absence of Ska1 loop. This was assessed by expressing an siRNA-resistant Ska1Δloop-GFP construct, in which the loop 92-132 region was deleted (Fig. 1*B*), in HeLa cells under depletion of endogenous Ska1 by siRNA (Fig. 1, *C* and *D*). The results were compared with full-length Ska1-GFP–expressed cells in parallel. While all the chromosomes in the Ska1-GFP cells could align to the metaphase plate completely, the Ska1Δloop-GFP failed to rescue metaphase alignment of a majority of the chromosomes (Fig. 1*C*). In the Ska1 Δloop-GFP cells, a large subset of chromosomes, though could localize at the spindle midzone, appeared to be aligned only partially to the metaphase plate and the rest appeared to be highly scattered. Under similar condition, the full-length Ska1-GFP–expressed cells displayed proper chromosome alignment in majority (∼78%) of the cells. Ska1Δloop-GFP expression induced chromosome misalignments in ∼88% mitotic cells (Fig. 1*E*). Broadly, three classes of chromosome misalignment defects, Class I, II, and III, were observed based on the severity of the defects (Experimental procedures) (Fig. S1*A*) and the sum of the percentages of all three was plotted. Consequently, Ska1Δloop-GFP failed to localize to the KTs (Fig. 1*F*). Same was evident from the intensity plot of KT-localized Ska1-GFP *versus* Ska1Δloop-GFP (Fig. 1*G*). The results infer that Ska1 loop is required for chromosome alignment to the metaphase plate and KT localization of Ska1.

### Ska1 interacts with EB1 through the loop and its KT recruitment requires EB1

**Figure 2:**
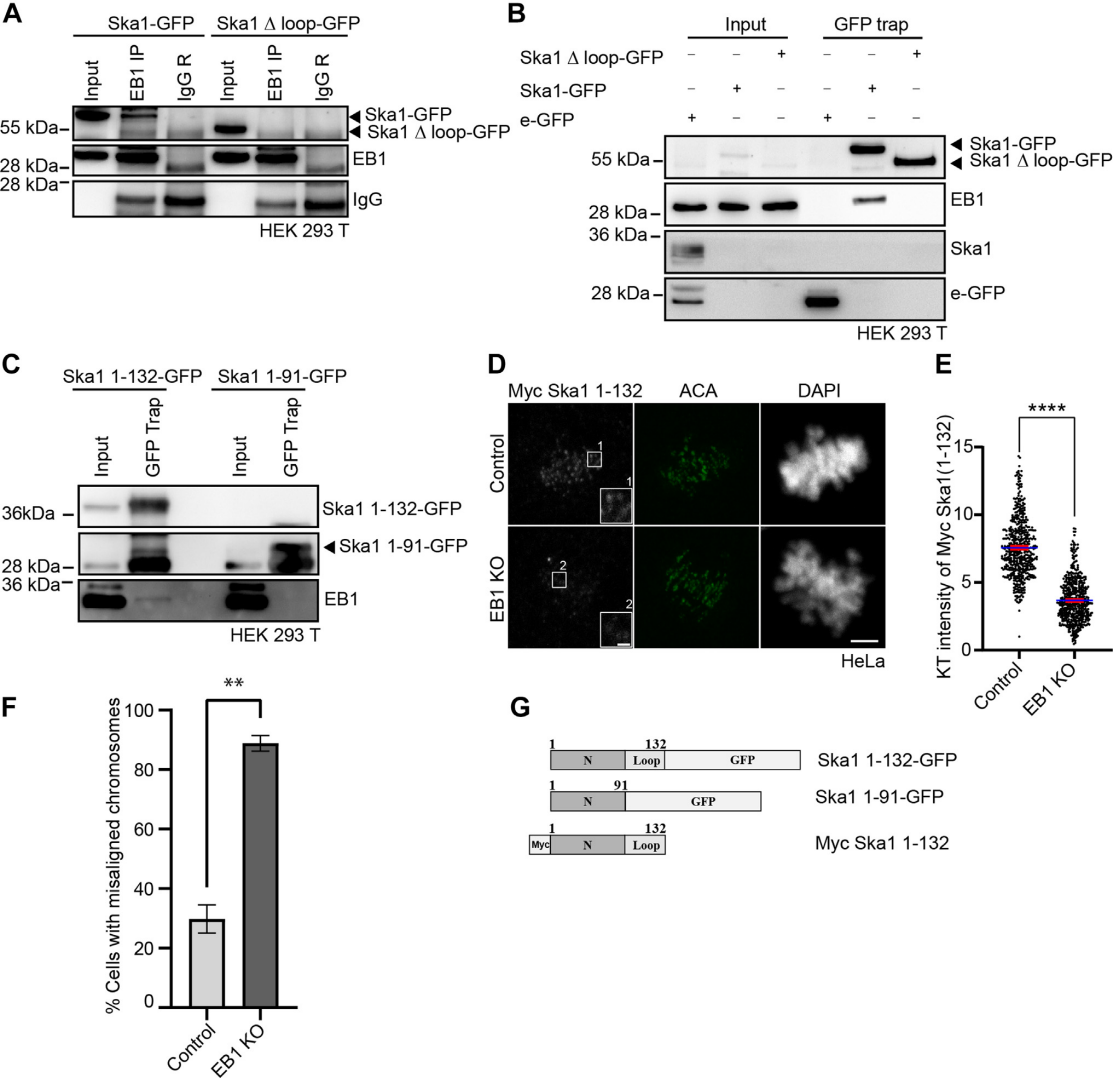
**Ska1 interacts with EB1 through its loop and its KT localization requires EB1.***A*, Ska1-GFP– and Ska1 Δloop-GFP–transfected HEK293T cells were mitotic synchronized by double thymidine and subjected to immunoprecipitation (IP) by using EB1 antibody, and the samples were analyzed for the presence of the Ska1 proteins by Western blotting. *B*, double thymidine–synchronized mitotic lysates of Ska1-GFP– and Ska1 Δloop-GFP–transfected HEK293T cells treated with Ska1 siRNA were immunoprecipitated using GFP trap beads followed by Western blotting to probe the presence of EB1. *C*, GFP-tagged Ska1 1-132– and Ska1 1-91–expressed HEK293T cells were synchronized, and the mitotic cell lysate was subjected to immunoprecipitation by using GFP trap beads. The presence of EB1 and GFP-tagged Ska1 proteins were detected by Western blotting. Rabbit or mouse IgG, wherever applicable, was used as control in all these experiments. *D*, representative immunofluorescence images of inducible EB1 knockout HeLa cells that were transfected with Ska1 siRNA and Myc-Ska1 1-132. Insets 1 and 2 represent Myc-Ska1 1-132 levels at the kinetochores in control and EB1 knockout cells, respectively. The scale bars in main and inset figures are 5 μm and 1 μm, respectively. EB1 knockout (EB1 KO) was induced by treating cells with doxycycline for 4 days. Control (without doxycycline) and EB1 KO cells were stained with Myc monoclonal antibody and polyclonal antibody against ACA. DNA was stained with DAPI. *E*, intensity of Myc-Ska1 1-132 localized at individual KT in control *versus* EB1 KO cells. Approximately, 500 KTs from three different experiments were counted in each case. ∗∗∗∗ refers to *p*< 0.0001. *F*, plot showing percentage of mitotic cells with misaligned chromosomes in Ska1 siRNA + Myc Ska1 1-132 in control *versus* EB1 knockout condition. ∗∗ refers to *p* = 0.0014. *G*, cartoon representations of Ska1 1-132-GFP, Ska1 1-91-GFP, and Myc Ska1 1-132 constructs. KT, kinetochore.

EB1 is essential for KT localization of Ska1 and it interacts with Ska1 (31). We therefore asked whether the Ska1 loop is involved in Ska1–EB1 interaction, which in turn can promote KT recruitment of Ska1. We first examined the role of Ska1 loop in Ska1-EB1 binding. While the immunoprecipitation of endogenous EB1 in HEK293T cells showed presence of the full-length Ska1-GFP, the Ska1Δloop-GFP did not show its detectable presence in the IP (Fig. 2*A*). Similarly, reverse pulldown of Ska1Δloop-GFP by GFP trap did not show any presence of endogenous EB1 in the lysates of Ska1Δloop-GFP–expressed cells under endogenous Ska1 knockdown by Ska1 siRNA; though under similar condition, the pulldown of full-length Ska1-GFP showed strong presence of EB1 (Fig. 2*B*). Therefore, Ska1 loop is essential for Ska1-EB1 binding.

Ska1 (1-132), consisting of the KT-targeting structural domain (1-91) followed by the loop, can localize to KTs (5, 32, 43), whereas we found that the structural domain of Ska1 (1-91) fails to localize to KTs in HeLa cells (Fig. S1*B*). It was also observed that Ska1 (1-132), but not Ska1 (1-91), interacts with EB1 (Fig. 2*C*). Though the amount of EB1 associated with Ska1 1-132-GFP as revealed by GFP nanobody-conjugated bead (GFP trap) method showed relatively less presence of EB1 as compared to EB1 associated with the full-length Ska1-GFP, possibly due to some interference caused by the GFP nanobody-fused bead (compare Fig. 2, *C* and *B*), the IP of Ska1 1-132-GFP by using GFP antibody showed a strong presence of EB1 (Fig. S1*C*). A reverse IP using EB1 antibody also showed strong presence of Ska1 1-132 GFP (Fig. S1*D*). Since the loop is essential for EB1 binding, we then checked whether KT recruitment of Ska1 1-132, which contains the loop, requires EB1. Myc-Ska1 1-132 was expressed in CRISPR-Cas9–based EB1 conditional knockout (EB1 KO) HeLa cells under doxycycline treatment (Experimental procedures) and its KT localization was assessed as compared to the control HeLa cells without doxycycline. As expected, the doxycycline-treated cells showed robust loss of EB1 expression (44) (Fig. S1, *E* and *F*). KT localization of Myc-Ska1 1-132 was substantially impaired in the EB1 KO cells as compared to control (Fig. 2*D*). The insets show better visualization of the differences of KT localized Myc-Ska1 1-132 (Insets Fig. 2*D*). Intensity analysis of individual KTs showed significantly reduced level of Myc-Ska1-1-132 in the EB1 KO cells (Fig. 2*E*). To rule out the possibility of any influence of altered MT dynamics due to EB1 knockout, if any, on KT localization of Ska1, we imaged HeLa cells expressed with Myc Ska1 1-132 in the presence of 300 nM nocodazole, which affects MT dynamics (45). KT localization of Myc Ska1 1-132 appeared to be minimally affected in the nocodazole-treated cells compared to its absence (Fig. S1, *G* and *H*). Consequently, chromosome misalignment defects were induced in the Myc-Ska1 1-132–expressed EB1 KO cells. Approximately, 88% mitotic cells had misaligned chromosomes in the Myc Ska1 1-132–expressed EB1 KO cells; whereas only 29% Myc Ska1 1-132–expressed control cells showed the misalignment defects (Fig. 2*F*). Together, the results indicate that binding of Ska1 loop to EB1 is essential for KT recruitment of Ska1.

### Conserved motif in Ska1 loop is critical for EB1–Ska1 interaction and metaphase chromosome alignment

**Figure 3:**
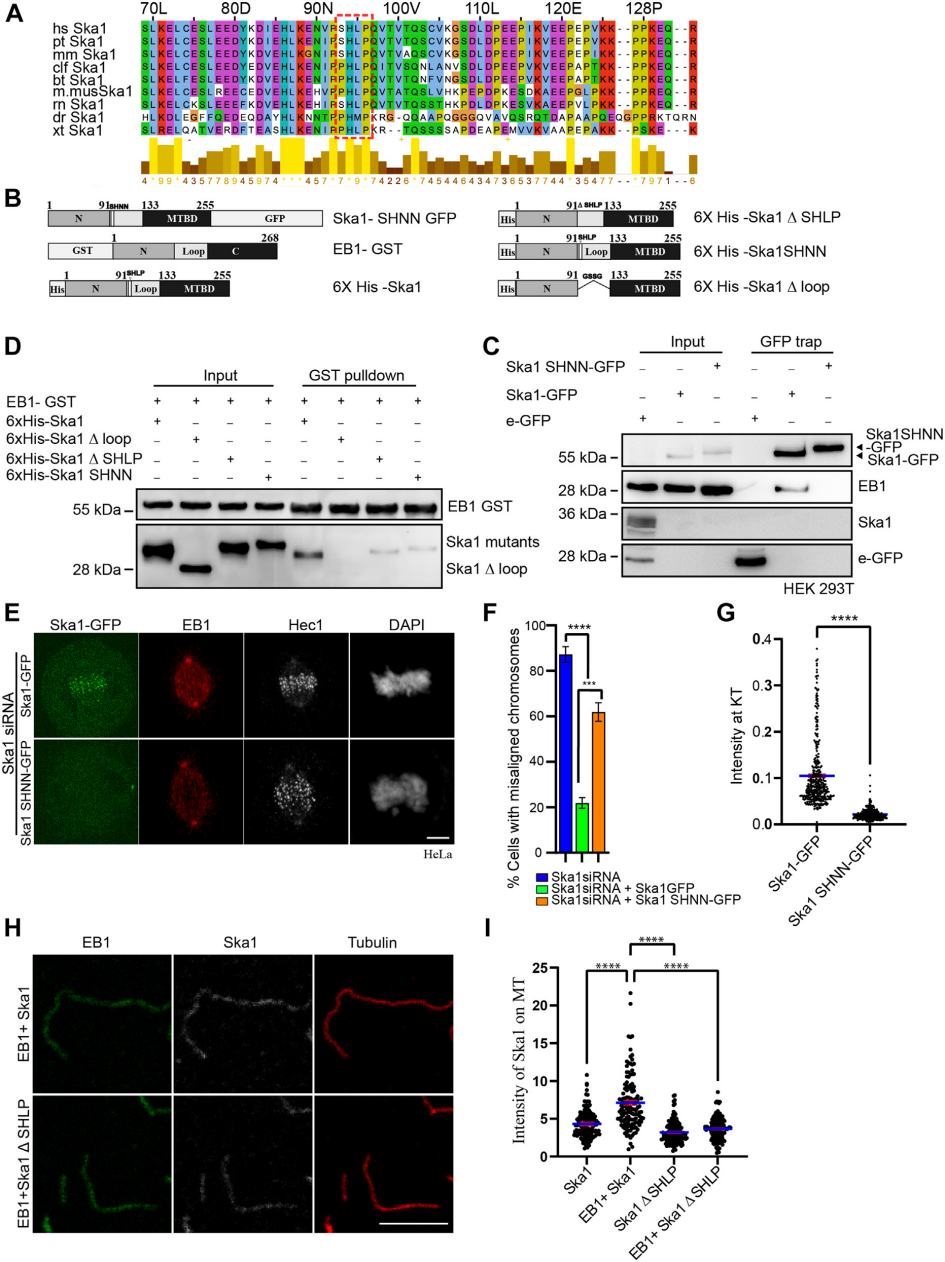
**SHLP motif of Ska1 is essential for its kinetochore localization and chromosome alignment in cells.***A*, amino acid sequences of SHLP-motif containing Ska1 region of humans (hs), *Pan troglodytes* (pt), *Macaca mulatta* (mm), *Canis lupus familiaris* (clf), *Bos taurus* (bt), *Mus musculus* (m. mus), *Rattus norvegicus* (rn), *Danio rerio* (dr), and *Xenopus tropicalis* (xt). *Dotted region* represents the SHLP or SHLP-like motifs in the proteins. The bars represent the conservation scores of the amino acids in the species. Scale 1 to 10 (represented as ∗). *B*, schematic representations of EB1-GST, WT Ska1, and various Ska1 mutant constructs. *C*, double thymidine–synchronized mitotic cell lysates of Ska1-GFP– and Ska1 SHNN-GFP–transfected HEK293T cells were immunoprecipitated using GFP trap beads followed by Western blotting to probe EB1. *D*, mixture of recombinant 6xHis-tagged Ska1 or Ska1 Δ loop or Ska1 ΔSHLP or Ska1 SHNN with EB1-GST was subjected to GST pulldown, and the association of EB1-GST with Ska1 WT *versus* Ska1 mutant proteins was probed by Western blot analysis of the pulldown samples. *E*, representative confocal images of Ska1 siRNA-transfected HeLa cells were expressed with Ska1-GFP or Ska1 SHNN-GFP for 48 h prior to staining with EB1 rabbit antibody and Hec1 mouse antibody. GFP-tagged proteins were imaged directly. DNA was stained with DAPI. *F*, plot showing the percentage of mitotic cells with chromosome alignment defects in the Ska1 WT *versus* Ska1 SHNN-GFP–expressed cells. Approximately, 60 to 80 mitotic cells counted in each experiment (no of experiments = 3 for each). Data are mean ± SEM. *G*, plot shows the intensity of KT-localized Ska1-GFP and Ska1 SHNN-GFP normalized to Hec1 in Ska1-GFP and Ska1 SHNN-GFP–expressed cells, respectively. Approximately, 300 KTs from three experiments were analyzed in each case. *H*, Ska1 or Ska1 ΔSHLP (0.5 μM) was added to the prepolymerized MTs *in vitro* in the presence or absence of EB1 (1 μM) and then stained with antibodies against α-tubulin, Ska1, and EB1. *I*, intensity plot of Ska1/Ska1 ΔSHLP localized onto MTs in the presence and absence of EB1. Number of MTs analyzed ∼120 in each case. ∗∗∗ refers to *p* = 0.0003; ∗∗∗∗ refers to *p* < 0.0001, ∗*p* < 0.05 (n = 3). Scale bar in all images represents 5 μm. MT, microtubule; KT, kinetochore.

Several +TIPs are recruited to MT plus ends by binding to EB1 through their conserved SXIP motif. Mutation of the hydrophobic IP of SXIP motif to NN (SHNN) disrupts EB1 binding and their MT plus end recruitment (36), emphasizing crucial role of the hydrophobic moiety provided by the residues I and P of SXIP for their EB1-dependent plus end recruitment. Human Ska1 consists of a similar motif with sequence SHLP in the upstream region in its loop and the motif is conserved in several vertebrates (Fig. 3*A*). We investigated possible role of this motif in EB1 binding by mutating LP of the motif to NN (Fig. 3*B*) (36). Pull-down of WT Ska1-GFP in HEK-293 cells depleted of endogenous Ska1 showed the presence of EB1, but not in the pull-down Ska1-SHNN-GFP, supporting the essential role of Ska1 SHLP motif for Ska1–EB1 interaction (Fig. 3*C*). This conclusion was further strengthened by GST pull-down using GST-tagged EB1, which could efficiently pulldown purified recombinant 6xHis-tagged WT Ska1, but not 6x His Ska1 SHNN or Ska1 ΔSHLP (the whole motif deleted version). Thus, WT Ska1 could associate with EB1-GST strongly, but the same was impaired drastically both in the case of Ska1 SHNN mutant and Ska1 ΔSHLP (Fig. 3*D*). As expected, 6xHis Ska1Δloop also did not show any association with EB1 GST. Previous studies developed a SXIP peptide aptamer (ALNGQSRIPVLKRHTR) that binds to EB1 strongly and interferes with plus end targeting of several EB1-binding proteins (46, 47). Therefore, we also checked if the EB1-binding SXIP peptide aptamer interferes with Ska1–EB1 interaction. GST pull-down assay showed that the peptide aptamer inhibits 6xHis-Ska1 binding to EB1-GST in a dose-dependent manner. At 1:5 M ratio of EB1:peptide, EB1-Ska1 association was reduced by ∼70% (Fig. S2*A*).

Next, we examined if Ska1 SHLP to SHNN mutation affects KT recruitment of Ska1. Expression of Ska1-SHNN-GFP under depletion of endogenous Ska1 (Fig. S2*C*) resulted in metaphase chromosome misalignments in ∼60% mitotic cells (Fig. 3, *E* and *F*). KT localization of Ska1 was also substantially reduced in the Ska1-SHNN-GFP–expressed cells (Fig. 3*G*). Similar defects were observed in cells expressed with the SHLP-deleted Ska1 variant (Ska1-ΔSHLP-GFP) (Fig. S2*B*). We also found that SHNN mutation does not affect Ska1 binding to Ska3 since the GFP pulldown from Ska1-GFP or Ska1-SHNN-GFP–expressed cells show Ska3 association to similar extent, suggesting that Ska complex assembly is not affected by SHNN mutation (Fig. S2*D*). However, KT localization of Ska3 was also reduced significantly in the Ska-SHNN-GFP–expressed cells as compared to Ska1-GFP control cells (Fig. S2, *E* and *F*), indicating that KT localization of not Ska1 alone but of the whole Ska complex is impaired in the SHNN mutant condition. We next sought to determine the effect of the absence of SHLP motif on Ska1 localization on MTs. Purified 6xHis WT Ska1 or the SHLP motif-deleted Ska1 (Ska1 ΔSHLP) was added to prepolymerized MTs *in vitro* in the presence of EB1, and Ska1 localization on the MTs was assessed by fluorescence microscopy. WT Ska1 was localized on the MTs to a significantly greater extent than Ska1 ΔSHLP (about 1.6 folds) (Fig. 3, *H* and *I*), supporting that EB1 interaction with Ska1 SHLP motif facilitates Ska1 recruitment onto MTs.

### High-speed AFM imaging shows association of Ska1 with EB1 dimer through its loop

**Figure 4:**
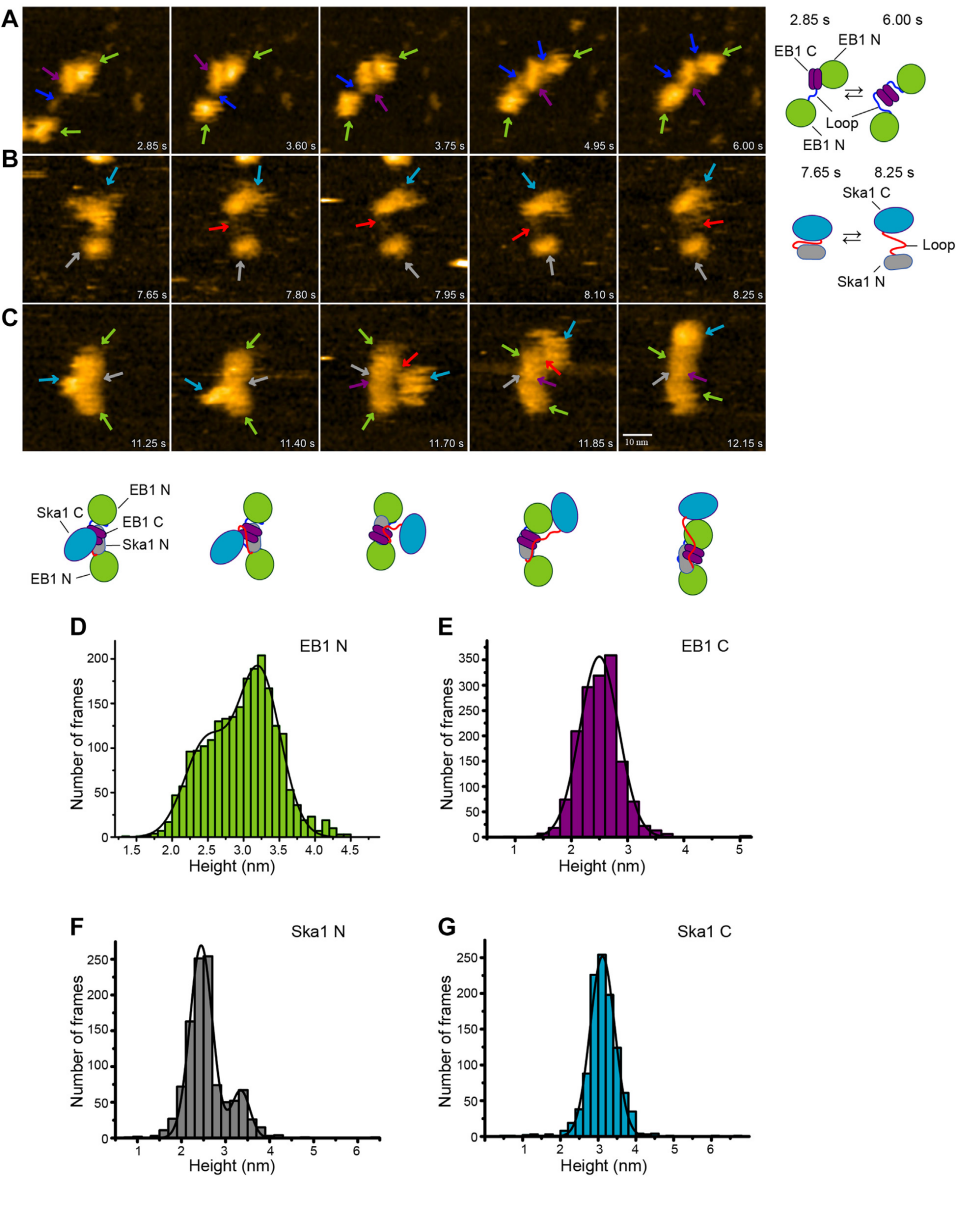
**Molecular dynamics of EB1, Ska1, and EB1–Ska1 complex.** HS-AFM images and schematic representations of EB1, Ska1, and EB1–Ska1 complex. *A*, HS-AFM images showing the dynamic changes of the domains of EB1 dimer (10 nM), (*B*) Ska1 monomer (10 nM), and *(C*) the same in the EB1–Ska1 complex (both 10 nM). The representative frames as shown are taken from the time lapse images (corresponding movies provided in Movies S1–S3) of EB1, Ska1, and EB1–Ska1 complex, respectively. Images were captured at 0.15 s per frame of 50 × 50 nm^2^ size at 80 × 80 pixel. The color scale was set at 4.25 nm for all the frames. The scale bar represents 10 nm. The schematics of different domains of EB1 and Ska1 were drawn freehand to represent the corresponding HS-AFM images. The arrowheads of different colors as shown denote the domains and loops of EB1 and Ska1 as per the schematics drawn. *D*–*G*, the plots represent the height distributions of EB1N, EB1C, Ska1N, and Ska1C domains with respect to number of frames. The lines in the plots are based on best fitting of the data. HS-AFM, high speed atomic force microscopy.

To visualize the interacting domains of Ska1 and EB1 during their complex formation in high resolution in real time, the dynamics of recombinant EB1, Ska1, and their mixture were imaged by high-speed atomic force microscopy (HS-AFM). This laboratory-built AFM integrated with a high-speed recording device (48, 49, 50) allowed capturing the domain organization and dynamics of the proteins at single molecule level within a time scale as fast as 150 milli-second per frame. Protein solutions were drop casted onto mica surface and the movements of the proteins were captured. EB1 protein alone was found to be majorly in the dimer form with its two N-terminal globular calponin homology domains (referred as EB1-N), separated from each other and a relatively flat and extended bar-like structure (referred as EB1-C), likely the EB1 C-terminal dimer (Fig. 4*A*). Rapid dynamics of the EB1 N and C domains was observed (Movie S1). Identity of EB1-N and EB1-C domains was confirmed from their average heights in the AFM images (Fig. 4, *D* and *E*). The Gaussian plots of height of EB1-N and EB1-C *versus* the number of frames with the corresponding heights are shown in Figure 4, *D* and *E*. The maximum height that was displayed in majority of the frames was considered as the average height of the respective domain. The heights of EB1 N and EB1 C as determined from the AFM data were consistent with their crystal structures (Fig. S3, *D* and *E*) (36, 42, 51, 52, 53). The AFM images additionally revealed the structural organization of the linker region connecting two EB1 N domains and EB1 C and it appeared as a flexible thin linker. Notably, a rapid length change ranging from ∼0 to 24 nm and concomitant stretching of the N-domains from the C-terminal dimer domain were evident (Movie S1). Majority (∼70%) of the frames displayed an average stretching length between ∼4 to 12 nm (Fig. S3*B*). HS-AFM imaging of Ska1 revealed that it exists exclusively in the monomer form, with its large C-terminal globular domain, a slightly extended N-terminus and the loop region connecting the two structural domains (Fig. 4*B*). Ska1 structural domains also exhibited dynamic stretching from each other, likely due to the connecting loop region (Movie S2). Although the two structural domains showed a minimum 2 nm and maximum 16 nm stretching, about 70% of the frames showed an average oscillation between 6 to 13 nm distance (Fig. S3*C*). The individual domains of Ska1 (Ska1 N and Ska1 C) were identified based on their maximum height analysis in the AFM images (Fig. 4, *F* and *G*). Ska1 C domain was identified by crossverifying the average length from its crystal structure of Ska1C (18, 43) with the maximum heights measured from the AFM images (Fig. S3*F*). Images of the mixture of EB1 and Ska1 showed formation of slightly curved extended structures with average length ∼30 to 35 nm (Figs. 4*C* and S3*A*). Ska1 appeared to be anchored to the bar-like coiled-coil EB1 C dimer structure through its flexible loop, while the globular Ska1 C region moves around the globular EB1 N domains and then positions itself vertically resulting in an extended structure with a slight curvature (Fig. 4*C* and Movie S3). Several such molecules with the similar structural organization were observed (Fig. S3*A*).

### Ska1 motif binds to residues in EB1 that are targeting sites of +TIPs

As SHLP motif in Ska1 loop is critical for EB1-mediated Ska1 recruitment to KTs and Ska1–EB1 interaction, we sought to identify the residues in EB1 that make contacts with the SHLP motif region of Ska1 by NMR. We studied the interaction between EB1 and a synthesized Ska1 peptide, Ska1 p with amino acid sequence KENVPSHLPQVTVT, which consists of the SHLP motif and its flanking region in both sides spanning from residues 88-101. Peptide was synthesized using solid phase peptide synthesizer and purified using HPLC. Intense base peak at m/z value ∼1590 corresponding to the molecular weight of the peptide is shown in Fig. S4*B*. To probe the interaction between the Ska1 SHLP peptide, Ska1 p, and EB1, ^15^N-^1^H TROSY of 2H-^15^N–labeled EB1 was measured with increasing concentrations of Ska1 p. After each addition of the peptide, pH was adjusted to 6.8. The overlaid spectra of EB1 in the presence and absence of Ska1 p showed a significant dose-dependent chemical shift changes of specific amino acids located within the EB homology (EBH) domain (residues 210-260 of human EB1) (36) of EB1 upon addition of Ska1 p (Figs. S5, *A*–*F* and S4*A*).

**Figure 5:**
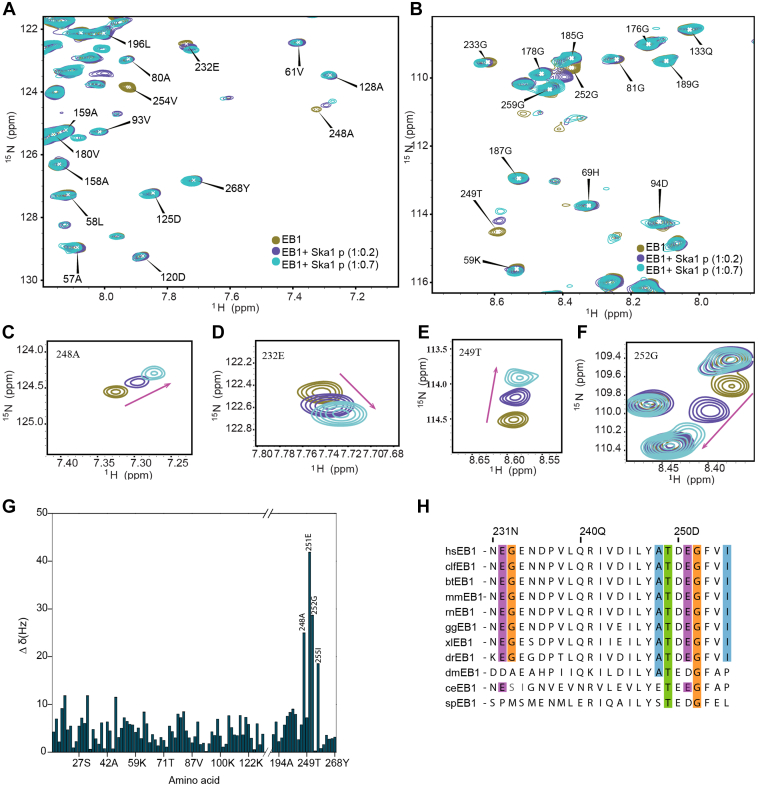
**Ska1 SHLP peptide binds to amino acids in EB1 C-terminus.** Overlaid ^15^N-^1^H TROSY spectra of EB1 alone and in the Ska1 SHLP peptide (Ska1 p)-bound states. *A* and *B*, TROSY spectra ^15^N-^1^H–labeled EB1 (95 μM) only; ^15^N-^1^H EB1 (95 μM) plus Ska1 p (19 μM); 15N-^1^H EB1 (95 μM) plus Ska1 p (66.5 μM) are shown. A number of residues in the C-terminal region of EB1 showed significant changes in their chemical shift values. *C*–*F*, close-up views of the representative cross peaks with the large chemical shift changes of the amino acids, 248A, 232E, 249T, and 252G of EB1 are shown. *G*, chemical shift perturbations of the amino acids in EB1 based on the ^15^N-^1^H TROSY spectra of EB1 upon addition of Ska1 p (66.5 μM) are shown. *H*, conservation of the amino acids of EB1 from different species that were affected significantly upon Ska1 SHLP peptide binding.

The residues 248A, 255I, 249T, 252G, 251E, 233G, and 232E in the EBH domain of EB1 showed significant change in their chemical shift values, when bound to the Ska1 SHLP peptide (Figs. 5, *A*–*F*, and S4*A*). The chemical shift changes of all the amino acids that were affected by the Ska1 SHLP peptide binding is shown in Figure 5*G*. It is interesting to note that the amino acids affected by Ska1 p binding largely overlap with the binding sites of other +TIPs proteins including MACF, APC (36). The amino acids showing significant change in their chemical shifts upon binding to the Ska1 p were also mapped on the X-ray crystal structure of EB1 C-terminus (52) and compared with those of the SXIP peptide aptamer/Ska1 SHLP peptide bound to EB1 (Fig. S4, *C* and *D*). Interestingly, the residues affected by Ska1 SHLP peptide are nearly the same subset of amino acids that showed large chemical shift deviations upon binding of EB1 to the SXIP aptamer peptide (47) and they are conserved across several metazoan species (Fig. 5*H*). Furthermore, nearly the same subset of amino acids was previously shown by NMR to be involved in binding of EB1 with the SXIP motif of +TIP, MACF (36) (Fig. S4*E*). These results together demonstrate that Ska1 SHLP motif binds to unique sites in EB1 that are specific for the SXIP-type +TIPs.

## Discussion

Ska1 plays critical role in the formation of stable KT-MT end-on attachment and sister KT biorientation. It stabilizes Ska complex association with the outer KT by binding with Ska3 and also by mediating interaction with NDC80 primarily through its N-terminal coiled-coil domain (1–91) (5, 18, 24, 26, 43). However, Ska1 (1-91) alone fails to localize to KTs in cells, suggesting that MT binding of Ska1 is a prior event, which then allows the complex to establish connection with the outer KT. Though Ska1 has an intrinsic MT-binding site in its C-terminal globular domain (133-255), Ska1 binding to purified MTs induces destabilization of MTs by facilitating more curvature to the depolymerizing MTs (5). Additionally, previous studies and our data here (Fig. 2*D*) have shown that Ska1 without its MT-binding C-terminal domain can still be recruited to the KTs efficiently (32, 43, 54). All these observations indicate that less likely the intrinsic MT-binding region of Ska1, but additional molecular interactions involving other regulatory site of Ska1 could be attributed to Ska1/Ska complex stabilization at the MT-KT interface *in vivo*. Supportively, we have shown here that interaction between the N-terminal loop (92-132) of Ska1 with MT plus end–associated protein EB1 is required for Ska1 stabilization at the KTs and metaphase chromosome alignment (Fig. 1). Previous study by time-lapse imaging showed that absence of the Ska1 loop causes significant delay in anaphase progression and chromosome alignment defects specifically at the stage just prior to anaphase entry (43). The chromosomes appeared to be loosely congressed as they spread over on a wider region on both sides of the metaphase plate. Consistently, our data of fixed cell images of the metaphase-arrested cells also showed a similar chromosome organization defect in the absence of the Ska1 loop. Additionally, scattered organization of some chromosomes near the spindle pole area was also observed (Fig. 1*C*). Interestingly, our results also revealed that a conserved motif, SHLP in the Ska1 loop region is primarily involved in regulating interaction of Ska1 with EB1 (Fig. 3). Abrogation of interaction specific to the motif is sufficient to result in the chromosome alignment defects analogous to the functional abrogation of not only the Ska1 protein alone but of the whole Ska complex at the KT, since we showed that Ska1 SHLP motif mutation disrupts KT localization of both Ska1 and Ska3 (Figs. 3 and S2). This functional impairment is not due to any defects in Ska complex assembly *per se*, since Ska1 binding to Ska3 was not affected upon mutation of the motif (Fig. S2). Previous studies showed that Ska complex localization to KT requires interaction of Ndc80 with Ska3 in a Cdk1-mediated Ska3 phosphorylation-dependent manner (26, 29). Here, our results indicate that the interaction of Ska1 with EB1 is essential for the KT recruitment of Ska complex. It is possible that both mechanisms are involved in this process. Since LP to NN mutation in Ska1 loop impairs both EB1 binding and KT localization of Ska1 (Fig. 3), and this mutation site is outside the Ska3-binding region, a likely possibility is that Ska1 interaction with EB1 could facilitate Ska3 interaction to Ndc80/KT. The idea that EB1–Ska complex interaction on the MTs occurs prior to Ska interaction with the KT is also supported by the fact that Cdk1 sites-specific Ska3 phospho-deficient mutant can localize to the MTs but not to the KT (29). As the sequence of the Ska1 motif bears close similarity with the general EB1-binding SXIP motif of several MT plus end–targeting +TIPs and the SHLP motif binds to residues in EB1 that the SXIP motifs of +TIPs bind to, it is reasonable to think that Ska1 is targeted to the KT-targeting MT plus ends *in vivo* in a similar way that other EB1-binding +TIPs do. This mechanism could be conserved across eukaryotes since the functional homolog of Ska in yeast, the Dam1 complex has also been shown to interact with yeast EB1, Bim1 through a similar motif (33).

Our AFM data enabled to visualize the dynamics of organization of different regions of EB1 and Ska1 proteins both individually and together during their complex formation. They also enabled to identify the structural regions of the two proteins that mediate interaction during their complex formation. The Ska1 molecule appeared to associate with the C-terminal dimer region of EB1 with its N-terminal flexible loop region during formation of the Ska1–EB1 complex (Fig. 4). While the loop being anchored, the structural domains of both the proteins reorganize in a manner that leads to formation of a slightly curved extended structure. Although the functional relevance of such unique structural organization is not clear at this time, it is likely that in this configuration, the MT-binding domains of the two proteins (two EB1 N calponin homology domains and one Ska1 C domain) are positioned towards one side. Such arrangement could favor increased MT binding of Ska1. Supportively, our data showed increased Ska1 localization onto purified MTs, when added together with EB1 (Fig. 3*H*). Majority of the Ska1-EB1 structures appeared to have slight curvature. We had shown previously that Ska1–EB1 complexes form distinct MT-bound structures *in vitro* and the structures were also curved in nature as they wrap around the MT lattice (31). However, unlike the EB1–Ska1 complex structures in the absence of MTs shown here, the MT-bound structures appeared more extended and they decorated nearly the whole MT lattice longitudinally (31). It is possible that the MT surface facilitates the formation of such extended structures by localizing many Ska1–EB1 complexes closely on the MT surface.

The SXIP-docking sites of nearly all the SXIP +TIPs lie within the EBH domain of EB1 C-terminus (36, 47, 55). Usually, the apolar I and P residues of SXIP are involved in packing interaction with the hydrophobic cleft of EBH and mutation of these sites to polar residues, such as Asparagine largely interferes with EB1 binding (36, 42). We showed that mutation of LP to NN in Ska1 SHLP abrogates Ska1–EB1 interaction and it leads to chromosome alignment defects (Fig. 3). Furthermore, Ska1 SHLP motif binds to the EBH domain, and the set of residues in EBH affected by Ska1 SHLP binding majorly overlaps with those involved in other key SXIP-type +TIPs binding (Figs. 5 and S4) (36, 42). Though the core SXIP motifs of +TIPs are indispensable for EB1 binding, the residues surrounding the motif also play important role to further facilitate EB1-binding. Usually, the motif is flanked in between an unstructured region rich with charged, more of basic residues and Serine residues (36, 41, 42). Ska1 SHLP motif is flanked between several basic amino acids (K82, H86, K88, K106, K117) and Serine residues (S76, S103, S108); and many of them are conserved (Fig. 3*A*). However, whether such unique sequence feature favors Ska1-EB1 binding remains to be tested in the future. Our results implicate that Ska1 recognizes EB1 on the MT plus ends through a molecular process that is analogous to the SXIP-type +TIPs. It is also to be noted that Ska1 is usually found to localize to the plus ends of the KT-targeting MTs, but not other types of MTs (5, 19, 31, 32). It is possible that binding with other proteins, such as Ska 2, 3, (18, 43, 56) and NDC80 (23, 26, 57), facilitate such KT MT-specific localization. KT MT-specific localization is also known for another EB1 binding +TIP, (MCAK) (58).

In conclusion, our results provide a mechanistic basis for the formation of stable MT-KT attachment during mitosis and revealed the involvement of a more general MAPs such as EB1 in regulating KT functions through site-specific interaction with the outer KT complex protein Ska1. It is important to note that function of Dam1 complex in yeast, the functional analog of metazoan Ska, is also regulated by EB1 (33) through a similar conserved motif of Dam1 protein, Duo1. These suggest a wider implication of our results. As EB1 is involved in organizing numerous plus end–targeting cargo proteins at the MT plus ends, a process that is likely to generate diverse plus end structures, it will be interesting to characterize the molecular details of those structures and their functional involvement in chromosome segregation.

## Experimental procedures

### Reagents and antibodies

Thymidine, DAPI, GTP, Pipes, nocodazole, and EGTA were obtained from Sigma. Dulbecco’s modified Eagle’s medium and fetal bovine serum (FBS) were purchased from Thermo Fisher Scientific. Tetracycline-free FBS was obtained from Cytiva. Mouse monoclonal antibodies against EB1 (Cat # 610534) and Actin (Cat# 612656) were obtained from BD Biosciences. Mouse monoclonal Myc (Cat# 66004-1-1g) and C-Myc (Cat# Sc-40) antibodies were obtained from Proteintech and Santa Cruz Biotechnologies, respectively. Rabbit polyclonal antibody of Ska1 (Cat # ab118586), Ska3 (Cat # ab186003), and rat monoclonal antibody of EB1 (Cat # ab53358) were obtained from Abcam. Rabbit polyclonal antibody for Ska1 (NBP1-72131) was obtained from Novus Biologicals. Mouse monoclonal antibody against α-tubulin (Cat # T6199) and the rabbit polyclonal anti-EB1 (Cat # E3406) and anti-GST (Cat # G7781) were obtained from Sigma. Mouse monoclonal antibodies of Hec1 (Cat # sc-135934) and CENP-A (GTX13939) were obtained from Santa Cruz Biotechnologies and Genetex, respectively. GFP antibody (Cat # 632381) was obtained from Clontech, Takara . GFP trap beads (gt-20) were obtained from Chromotech (Germany). The dilutions of the primary antibodies were EB1 (IF-1:1000 of Sigma, Abcam and WB-1:3000 of BD, Sigma), α-tubulin (IF- 1:700 and WB:-1:3000), Ska1 (IF-1:250 Abcam, WB- 1:500 Novus), Hec1 (IF-1:200), CENP-A (IF -1:200), and Myc (IF-1:300, WB-1: 500). Alexa fluor–conjugated donkey anti-mouse 488 and anti-rabbit 568 secondary antibodies were obtained from Invitrogen. Anti-rat TRITC, Anti-mouse Cy5, and peroxidase-conjugated secondary antibodies were obtained from Jackson Immuno Research.

### Cell culture and transfection

HeLa and HEK293T cells were grown in Dulbecco’s modified Eagle’s medium supplemented with 10% FBS, 2 mM L-glutamine, 1.5 mg/ml sodium bicarbonate, 100 μg/ml penicillin, and 100 μg/ml streptomycin in a humidified environment with 5% Co2. For depletion of endogenous Ska1, 60% confluent HeLa cells were transfected with Ska1 siRNA (5′-CCCGCTTAACCTATAATCAAA-3′) (Cat # D-015917-04) (31). For control, Sigenome siRNA (5′-GCCAUUCUAUCCUCUAGAGGAUG-3′) (Cat # D-001210-01-05) was used. For rescue experiments, siRNA-resistant GFP-tagged Ska1 WT and various mutant variant plasmid DNA were transfected after 12 h of siRNA treatment. Cells were treated with MG132 (25 μM) for 2 h prior to collection 48 h post transfection of the plasmid DNA. Inducible Cas9 HeLa cells stable for specific EB1 guide RNA were obtained from Iain Cheeseman (Whitehead Institute). The cells were maintained in growth medium containing 10% tetracycline-free FBS. For generating EB1 knockout, Cas 9 was induced by treating the cells with 2 μg/ml doxycycline at 24 h interval for 4 days prior to analysis of EB1 protein level (44). For rescue experiments with Myc-Ska1 1-132 GFP, the cells after 2 days of doxycycline treatment were transfected with Ska1 siRNA (12 h) followed by transfection of the plasmid DNA while continuing the doxycycline treatment for the next 2 days prior to analysis. For assessing KT localization of Myc-Ska1 1-132 in the presence of nocodazole, HeLa cells transfected with Myc Ska1 1-132 under endogenous Ska1 depletion were synchronized by double thymidine (2mM) and treated with 300 nM nocodazole (6 h after thymidine release) for 4 h prior to fixing and staining. Lipofectamine RNAimax (Invitrogen Life Technologies) was used as vehicle for transfection of siRNA, and lipofectamine 3000 was used for plasmid DNA transfection.

### Plasmids and proteins

Ska1-GFP construct was made by PCR amplification of WT Ska1 from pIC291plasmid and cloned in to pcDNA3-EGFP vector having CMV promoter. The construct was made SiRNA resistant by using site-directed mutagenesis. Ska1 1-132 GFP as well as Myc-tagged constructs were made by PCR amplification of 1-132 region from Ska1 siRNA resistant construct and was subcloned into pcDNA3-EGFP and PCMV-Myc vectors, respectively. WT Ska1-GFP plasmid used in this study was generated using the pIC291 Ska1-GFP (from Iain Cheeseman lab, Whitehead Institute) as template for PCR amplification of the coding sequence of the human Ska1 gene. The amplified product was ligated into pcDNA3-EGFP (Novagen) for WT Ska1-GFP. The construct was made Ska1 siRNA-resistant by site-directed mutagenesis. Ska1 Δloop-GFP construct was generated by PCR amplification of the Ska1 region devoid of loop (residues 92-132) and connected from an siRNA-resistant PCDNA3.1 Ska1 Δloop-mCherry construct (a gift from A. Jeyaprakash, University of Edinburg) followed by cloning into a pcDNA3-eGFP vector. The N (1-91)- and C-terminal (133-255) regions were connected through a short peptide (GSSG) in the Ska1 Δloop-mCherry construct (43). Ska1 SHNN-GFP and Ska1 ΔSHLP-GFP plasmids were generated by site-directed mutagenesis from the WT Ska1 GFP plasmid. Ska1 1-132 GFP and Myc Ska1 1-132 were generated by PCR amplification of 1-132 region of the WT Ska1-GFP siRNA-resistant construct and was subcloned into pcDNA3-EGFP and pCMV-Myc vectors, respectively. His Ska1-GFP construct was made by PCR amplification of the Ska1-GFP region from pcDNA3 Ska1-GFP plasmid and subcloned into pET28a vector. For His Ska1 1-132-GFP, the GFP-tagged 1-132 Ska1 region from pcDNA3 EGFP vector containing Ska1 1-132-GFP was PCR amplified and subcloned into pET28a vector.

For GST-tagged EB1, EB1 cloned into a pGEX 5 × 3 vector was used (31). For obtaining 6xHis Ska1 WT protein, pEC-S-CDF-His Ska1 (gift from A. A. Jeyaprakash, Wellcome Trust Centre for Cell Biology) plasmid was used. The His-tagged Ska1 SHNN- and Ska1-ΔSHLP constructs generated from the pEC-S-CDF-His Ska1 through site-directed mutagenesis. His-tagged Ska1 Δloop construct was generated by PCR amplification of the Ska1 Δloop region from the Ska1 Δloop-mCherry construct and then subcloned into a pET28a vector. All the His- and GST-tagged plasmids were expressed in *Escherichia coli* BL21 DE3 cells, and the proteins were purified using Ni^2+^-NTA (Qiagen,) and Glutathione Sepharose (GE Healthcare). The purified proteins were stored at −80 °C. Protein concentrations were estimated using Pierce BCA (Bicinchoninic acid) protein assay kit (Thermo Fisher scientific).

### Immunofluorescence microscopy and image analysis

Cells after fixing in methanol at −20 °C were washed with PBS containing 2% bovine serum albumin and 0.5% Triton X-100. The cells were then incubated with primary antibody for 2 h followed by incubation with secondary antibody and DAPI for 60 and 1 min, respectively. Coverslips were mounted using ProLong Gold (Invitrogen), and the images (63×) were captured using a Leica SP5 laser confocal microscope. The intensity per pixel of Ska1-GFP WT or mutant proteins per KT was measured by selecting regions of interest of fixed area around the KT after background subtraction using Image J Fiji software (https://imagej.net/software/fiji/downloads). Chromosome misalignment defects in all cases were quantified in a similar way as previously described (5). Briefly, the defects were classified broadly into three classes, Class I, II, and III. Class I: a significant number of chromosomes could align at the metaphase plate with a few misaligned chromosomes. Class II: majority of the chromosomes are misaligned and poorly congressed. Class III: severely misaligned chromosomes with multipolar spindles. The sum of class I, II, and III represents the total percentage of mitotic cells with congression defects.

### Co-IP and GST pull-down assays

Cells were mitotically synchronized using double thymidine block. EB1 was immunoprecipitated from the mitotic cell lysates using EB1 antibody (59). For pull-down of Ska1-GFP or Ska1 mutant-GFP proteins using GFP-trap (Chromotech), the manufacturer’s protocol was followed. Briefly, the cell lysates were incubated with the equilibrated GFP-trap beads for 4 h and then the proteins bound to the beads were analyzed after washing the beads followed by lysing with sample buffer. Coimmunoprecipitation of Ska1-GFP proteins was performed using GFP antibody (Takara Bio).

Protein A/G beads were used for antibody-based coimmunoprecipitation. *In vitro* GST pull-down was performed by incubating purified Ska1 WT or mutant proteins with EB1-GST preincubated with GSH-Sepharose beads. The beads were washed with lysis buffer and then boiled in SDS-PAGE sample buffer for immunoblot analysis. For the experiments with peptide aptamer, EB1-GST protein was preincubated with the aptamer for 2 h and then the GSH beads were added. The bead solutions were incubated with the Ska1 WT protein and the pull-down assay was performed.

### Atomic force microscopy

HS-AFM images were acquired in an in-house–built AFM instrument equipped with high-speed recording device (50, 60, 61). Sample stage consists of a mica sheet of 1.5 mm diameter and ∼ 0.05 mm thickness attached onto a glass cylinder (2 mm height and 2 mm diameter) through epoxy glue. The mica-attached cylinder is sticked onto the Z piezo of the scanner by using nail polish. Freshly cleaved mica surface was prepared by peeling off the top layer of mica sheet using an adhesive tape. Two microliters of protein (EB1 or Ska1) in BRB80 buffer (80 mM Pipes, 1 mM EGTA, 1 mM MgCl_2_, pH 6.9) with varying concentrations (10–30 nM) were loaded onto the mica sheet and incubated for 3 min. The stage containing the sample was then rinsed with 20 μl of BRB80 buffer to remove the floating samples and then was immersed in a liquid cell containing the observation buffer. AFM images were then captured in tapping mode using small cantilevers (BL-AC10DS-A2, Olympus) (resonance frequency, ∼ 0.5 MHz in water, quality factor, ∼1.5 in water, Spring constant, ∼0.1 N/m). The cantilever’s free oscillation amplitude *A*_*0*_ and set-point amplitude *A_s_* were set at 1 to 2 nm and 0.9 ∼ 0.9 × *A*_*0*_, respectively. Images were captured at 150 milli-second per frame.

HS-AFM images were analyzed and refined by using laboratory-made software (50). Spike noise in the images was removed by applying a low pass filter. XY plane was flattened by using a flattening filter. The XY coordinate of the highest point on the protein domains was determined semi-automatically. First, a point likely to be the highest point was manually assigned and then, the software finds the exact maximum height point in a 5 × 5 pixels area surrounding the manually selected point. The height of individual domains was determined by subtracting the average height of the substrate from the highest identified in the same manner, and the distance between the individual domains was measured from the XY coordinates of the highest point.

### MT sedimentation assay

Tubulin (15 μM) was polymerized in BRB80 buffer (80 mM Pipes, 1 mM EGTA, 1 mM Mgcl2 pH 6.9) in the presence of 10% DMSO, 15 μM Taxol, and 1 mM GTP at 35 °C for 15 min. Aliquots of polymerized MTs were incubated with EB1 (1 μM) for 5 min followed by incubation with WT Ska1 or Ska1 ΔSHLP (0.5 μM) for another 15 min at room temperature. The MT-protein mixtures were then fixed with 1% glutaraldehyde in BRB80 for 5 min at room temperature followed by diluting 50 times in BRB80 buffer prior to layering on a 15% glycerol cushion and sedimentation onto 0.1% poly-L-lysine–coated coverslips. Coverslips were blocked with 1% BSA-BRB80 for 30 min and incubated with mouse monoclonal α-tubulin (Sigma), rat monoclonal EB1 (Abcam), and rabbit polyclonal Ska1 antibodies (Novus) for 45 min followed by incubation with secondary antibodies, anti-mouse Alexa 555, anti-rat Alexa 488, and anti-rabbit Alexa 647. Images were captured using Leica SP5 laser confocal microscope. The fluorescence intensities of the proteins associated with MTs were analyzed using Leica LAS AF lite software. The per-pixel intensity of Ska1 bound to MTs was quantified by drawing line region of interest of 2 μm length on the MTs (∼120 in number in three experiments) after background subtraction.

### Synthesis and purification of Ska1 peptide

Fmoc-based solid state peptide chemistry was used for the synthesis of Ska1 SHLP peptide (Ska1 p) (amino acid sequence-KENVPSHLPQVTVT) using PS3TM Peptide Synthesizer. Fmoc-protected amino acids and the reagents were purchased from Sigma Aldrich. Rink amide MBHA resin (Novabiochem) was used as the solid surface for the attachment of C-terminal amino acid of the peptide. Deprotection of the N-alpha position takes place next followed by the activation and coupling of the second amino acid. HBTU ((2-(1H-benzotriazol-1-yl)-1, 1, 3, 3-tetramethyluronium hexafluorophosphate) was used as the coupling agent. The steps were repeated till the last amino acid and acetic anhydride was used to acetylate the N-terminus of the peptide. Resin was then washed using dichloromethane and dried. Reaction using the cleavage cocktail (88% TFA, 5% phenol, 5% water and 2% tri-isopropyl silane) cleaved the peptide from the resin. Cleaved peptide was then precipitated in ice cold ether, dried, and dissolved in glacial acetic acid to lyophilize. Reverse phase HPLC (Agilent Technologies) was used to purify the peptide. Purity was confirmed using matrix-assisted laser desorption/ionization mass spectrometry. Pure peptide was then concentrated, washed, and lyophilized in water.

### NMR titration experiments

All the ^15^N-^1^H TROSY titration experiments were carried out in 700 MHz NMR spectrometer with 16 scans and 256 complex points. To determine the interaction between EB1 and Ska1 peptide, increasing concentrations of Ska1 starting from 0.5 μM to 65 μM was added to 95 μM 2H-^15^N–labeled EB1 in 50 mM potassium phosphate buffer containing 300 mM KCl, 1 mM DTT, and 10% D_2_O at pH 6.8. All the ^15^N-^1^H TROSY spectra were processed using NMRPipe (62) (https://www.ibbr.umd.edu/nmrpipe/install.html) and SPARKY (63) software (https://www.cgl.ucsf.edu/home/sparky/).

### Chemical shift perturbation

The combined change in chemical shift on ^15^N and ^1^H dimension was calculated using the equation.

Δδ = √ (Δδ*HN*) ^2^ + (0.17∗Δδ*N*) ^2^

where Δδ = combined chemical shift in Hz

Δδ*HN* = chemical shift change in the ^1^H dimension (Hz)

Δδ*N* = chemical shift change in the ^15^N dimension (Hz)

### Statistical analysis

Data are presented as mean ± SEM. The normally distributed data were analyzed with modified Student’s (Welch) *t* test at the 99% confidence level. Wherever applicable, one way ANOVA followed by Tukey’s multiple comparison tests were performed. The data were plotted and analyzed using Origin Pro 8.6 and GraphPad Prism 6 software. The figures were organized using Adobe Photoshop and Adobe Illustrator.

## Data availability

All relevant data are available.

## Supporting information

This article contains supporting information.

## Conflict of interest

The authors declare no conflict of interest.
