# Supplementary material for: Kinetochore-microtubule attachment in human cells is regulated by the interaction of a conserved motif of Ska1 with EB1
Source: J Biol Chem. 2022 Dec 31;299(2):102853. doi: 10.1016/j.jbc.2022.102853 (PMC9926122; doi:10.1016/j.jbc.2022.102853)
Supplement: Supporting information [file mmc4.pdf]

Supporting data

Fig. S1.

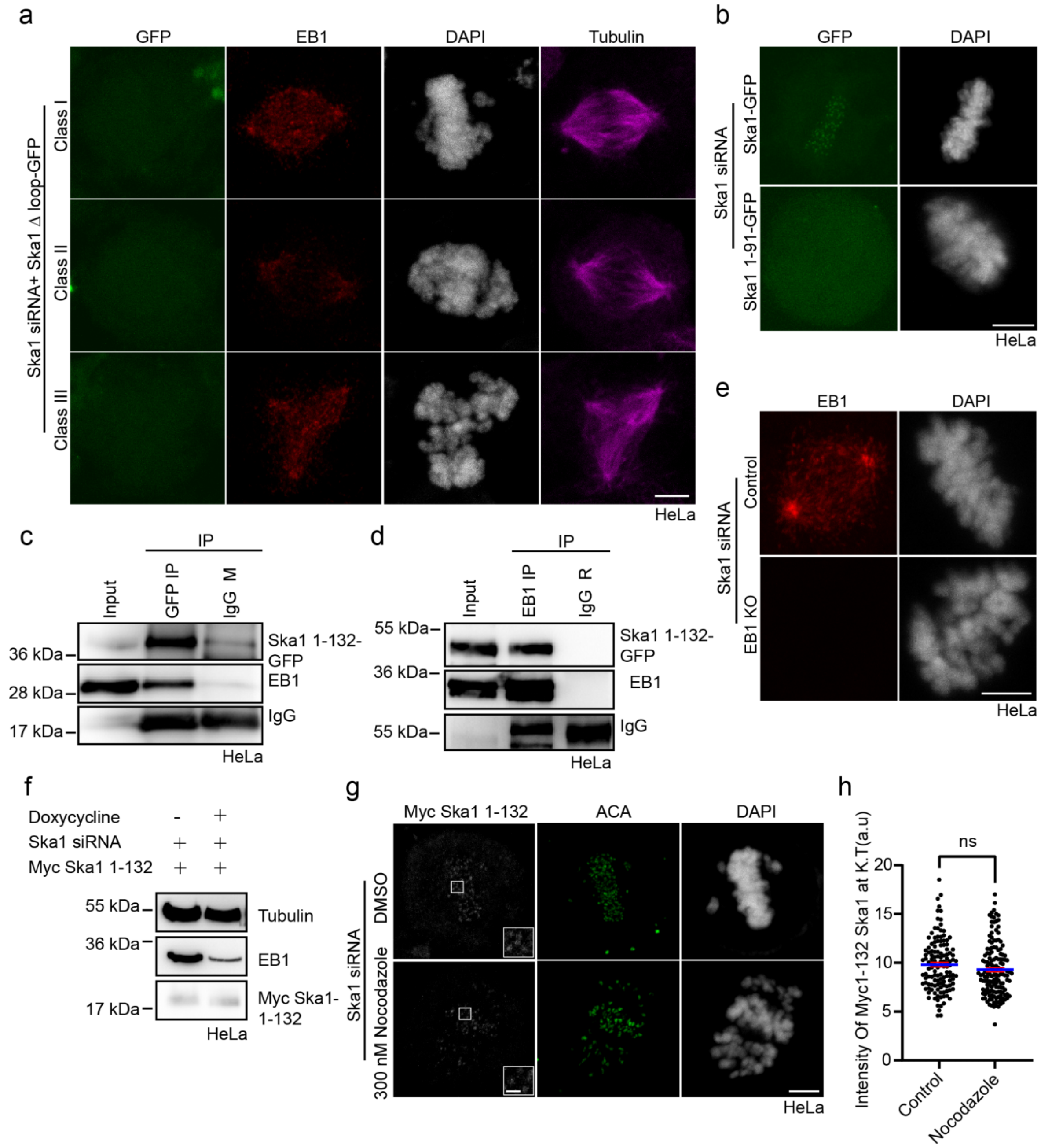

Figure S1. (a) Representative immunofluorescence images of Ska1Δ loop-GFP-expressed HeLa cells under endogenous Ska1 depletion showing the different classes of chromosome misalignment defects as Class I, Class II and Class III. (b) Representative immunofluorescence images of Ska1-GFP WT vs. Ska1 1-91-GFP-expressed HeLa cells showing no localization of Ska1 1-91-GFP at the KTs. (c) Ska1 1-132-GFP-expressed double thymidine synchronized

mitotic HeLa cell lysate was immunoprecipitated with GFP antibody followed by Western blotting to detect the presence of EB1. (d) Ska1 1-132-GFP-expressed HeLa cells were mitotic synchronized by double thymidine and EB1 was immunoprecipitated with EB1 antibody followed by detection of 1-132 Ska1-GFP in the immunoprecipitate by Western blotting. (e) Representative immunofluorescence images of inducible knockout HeLa cells targeting EB1. EB1 knockout (EB1 KO) was induced by treating the cells with doxycycline for four days. Control (without doxycycline) and EB1 KO cells were stained with EB1 antibody. (f) Western blot image showing expression levels of EB1 in control vs. EB1 KO HeLa cells. Expressions of exogenously expressed Myc Ska1 1-132 in these two conditions are also shown. (g) Representative immunofluorescence images of Myc Ska1 1-132-expressed HeLa cells under depletion of endogenous Ska1 by Ska1 siRNA showing kinetochore localization of Myc Ska1 1-132 under control (DMSO) vs. 300 nM nocodazole treatment. Cells were stained with Myc monoclonal antibody and polyclonal antibody against ACA. DNA was stained with DAPI as shown in all the microscopy image panels. The scale bars in the main and inset figures are 5 μm and 1 μm, respectively. (h) Plot showing the levels of Myc Ska1 1-132 at kinetochores upon 300 nM nocodazole treatment vs. control. ~100 kinetochores in each case were analysed from three experiments.

Fig. S2.

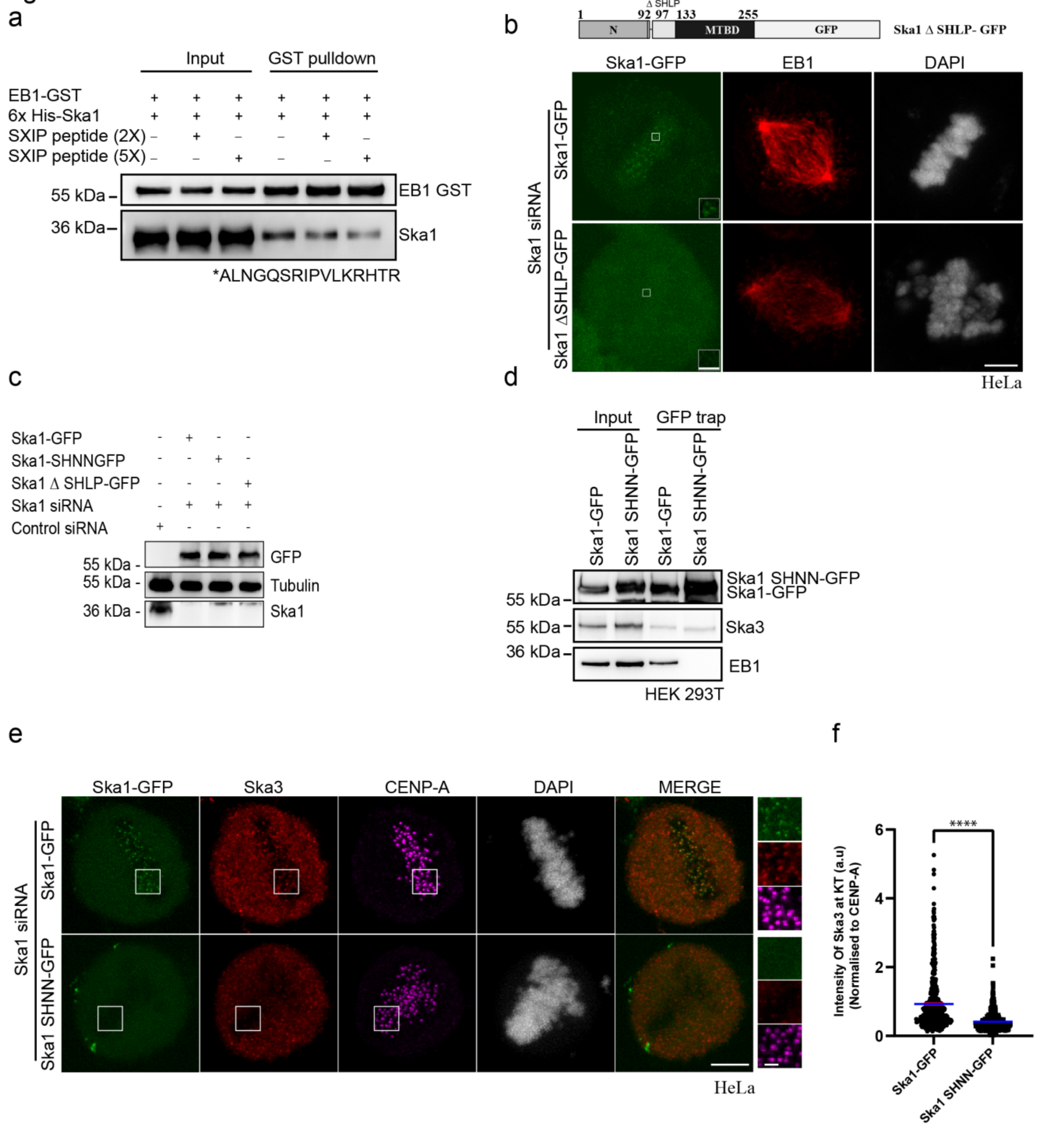

Figure S2. (a) EB1-GST was mixed with increasing amounts (2- and 5- folds molar excess) of the SXIP peptide aptamer and then the mixture was added to recombinant His-tagged Ska1 and the solutions were subjected to GST pull-down. Association of EB1-GST with 6xHis Ska1 in presence of the aptamer was assessed by Western blot of the EB1-GST and Ska1 proteins. (b) Representative immunofluorescence images of endogenous Ska1-depleted HeLa cells expressed with Ska1-GFP or Ska1ΔSHLP-GFP. Ska1ΔSHLP-GFP-expressed cells show chromosome alignment defects similar to the Ska1 SHNN-GFP-expressed cells. Ska1 ΔSHLP-

GFP also fails to localize to the MT-KT interface. The scale bars in the main and inset figures are 5 µm and 1 µm, respectively. (c) Western blot images showing the expression levels of Ska1 WT and mutant variants in the Ska1 siRNA-treated HeLa cells. Levels of endogenous Ska1 are also shown. (d) Double thymidine synchronized mitotic cell lysates of Ska1-GFP-and Ska1 SHNN-GFP-transfected HEK 293T cells were immunoprecipitated using GFP trap beads followed by Western blotting to probe for Ska3, EB1 and Ska1-GFP proteins. (e) Representative confocal images of Ska1 siRNA-transfected HeLa cells were expressed with Ska1-GFP or Ska1 SHNN-GFP for 48 hrs prior to staining with antibody against Ska3 and CENP-A. GFP-tagged proteins were imaged directly. DNA was stained with DAPI. Enlarged insets for kinetochore localization of GFP-tagged Ska1 proteins, Ska3 and CENP-A are shown. The scale bars in the main and inset figures are 5 µm and 1 µm, respectively. (f) Plot shows the intensity of KT localized Ska3 normalized to CENP-A in Ska1-GFP and Ska1 SHNN-GFP expressed cells, respectively. ~ 500 KTs from three experiments were analyzed in each case.

Fig. S3.

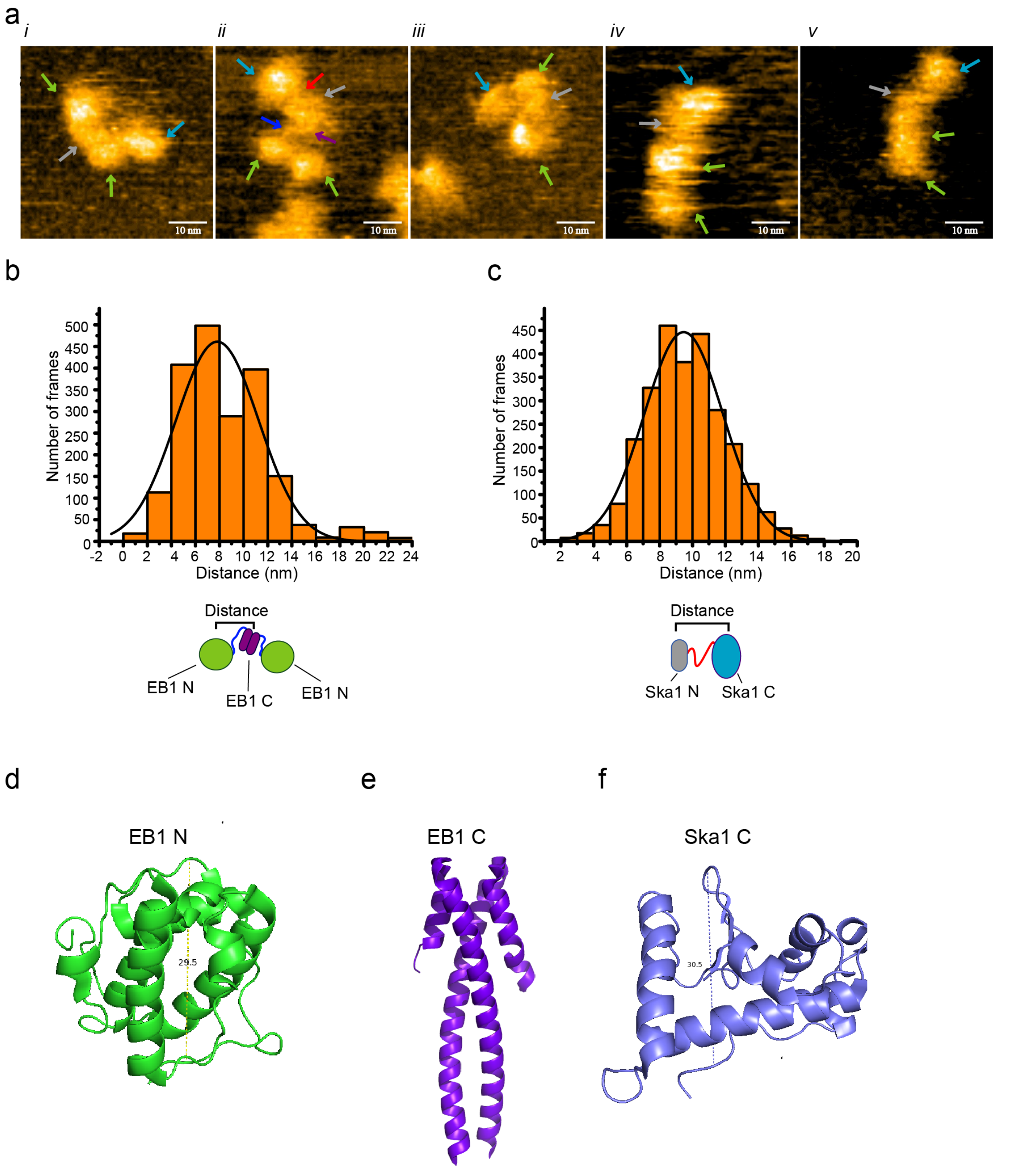

Figure S3. a) Molecular dynamics of EB1, Ska1 and EB1-Ska1 complex.

Additional HS-AFM images of EB1-Ska1 complexes of similar structural feature. EB1N, Ska1C and the Ska1 loop-containing region interacting with EB1 C region are shown by green, light blue and grey arrow, respectively. Ska1 loop and EB1 linker regions, wherever visible, are shown by red and dark blue arrows, respectively. b) and c) Plots represent the distribution

of the distances between EB1 N to EB1 C and Ska1 N to Ska1 C domains, respectively. d), e) and f) Cartoon representations of the crystal structures of EB1 N , EB1 C dimer and Ska1 C domains from PDB (EB1 N: 2R8U; 1YIG; Ska1 C: 4C9Y).

Fig. S4.

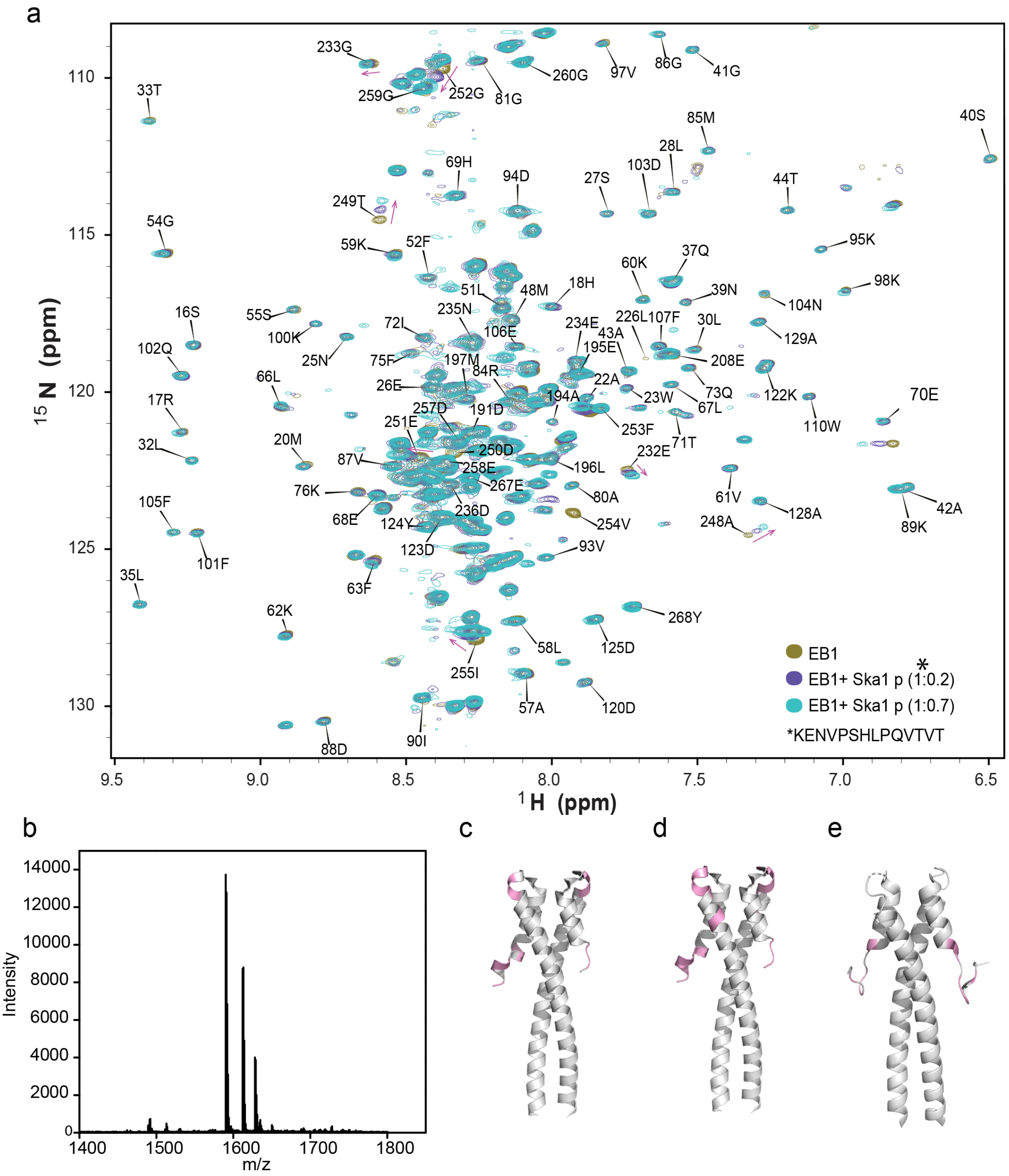

Figure S4. Ska1 SHLP motif interacts with EB-homology domain of EB1.

(a) Overlaid 15N-1H TROSY spectra of all the amino acids of EB1 alone and in the Ska1 SHLP peptide (Ska1 p)-bound state. EB1 and Ska1 p molar ratios were 1: 0.2 and 1: 0.7. Amino

acid sequence of Ska1 p is shown (denoted as *). (b) MALDI spectrum of Ska1 SHLP peptide, Ska1 p. Purity of the sample was confirmed by the intense peak corresponding to molecular ion. (c-e) The crystal structures of EB1 C-terminal dimer domain (1YIG.pdb) (c) Residues that showed significant changes in their chemical shift values on addition of Ska1 p are highlighted with colour. (d) Residues that showed large change in their chemical shift values on addition of SXIP aptamer are highlighted. (e) Residues of human EB1 in complex with microtubule plus end localization SXIP signal peptide of MACF are highlighted (3GJO.pdb).

Movie S1. Hs-AFM movie corresponding to the image frames shown in Figure 3a.

Movie S2. Hs-AFM movie corresponding to the image frames shown in Figure 3b

Movie S3. Hs-AFM movie corresponding to the image frames shown in Figure 3c
